# Basophil *FCER*1A and *PTAFR* Gene Expression Profiles Correlate With Disease Severity in Chronic Spontaneous Urticaria

**DOI:** 10.1002/clt2.70168

**Published:** 2026-04-17

**Authors:** Kevin Muliawan Soetanto, Chattip Sripatumtong, Teerapat Paringkarn, Nattha Angkoolpakdeekul, Kanokvalai Kulthanan, Yuttana Srinoulprasert

**Affiliations:** ^1^ Department of Immunology Faculty of Medicine Siriraj Hospital, Mahidol University Bangkok Thailand; ^2^ Department of Dermatology Faculty of Medicine Siriraj Hospital, Mahidol University Bangkok Thailand

**Keywords:** basophils, chronic spontaneous urticaria, *FCER*1A, *PAFR*, severity

## Abstract

**Background:**

Chronic Spontaneous Urticaria (CSU) is a debilitating skin condition characterized by recurrent wheals and pruritus, significantly impacting quality of life. Molecular mechanisms underlying different severity phenotypes are not fully understood. This study aimed to investigate the expression of *FCER*1A and *PTAFR* genes in basophils implicated in CSU pathogenesis from CSU patients with varying disease severities.

**Methods:**

We recruited 45 CSU patients, stratified into mild (*n*  =  15), moderate (*n*  =  15), and severe (*n*  =  15) groups, and 15 healthy controls. Basophils were isolated from peripheral blood, and the relative mRNA expression of *FCER*1A and *PTAFR* genes was quantified using real‐time PCR.

**Results:**

CSU patients exhibited significantly higher expression levels of *FCER*1A and *PTAFR* genes compared to healthy controls. *FCER*1A expression was significantly elevated in all CSU groups compared to controls and was higher in moderate and severe groups than in the mild group. *PAFR* expression was also significantly higher in moderate and severe CSU. Correlation analysis revealed that both *FCER1A* and *PTAFR* mRNA expression levels positively correlate with CSU severity.

**Conclusion:**

The expression of *FCER*1A and *PTAFR* genes in basophils correlates significantly with CSU severity, suggesting their potential as both prognostic and severity biomarkers. These findings highlight key molecular pathways that could be targeted for therapeutic intervention. Early detection of elevated gene expression could facilitate timely, targeted treatments, potentially reducing the progression to severe disease in CSU patients.

## 1 Introduction

Urticaria is classified based on its duration. Acute urticaria persists for 6 weeks or less, whereas chronic urticaria (CU) involves symptoms occurring at least twice a week for more than 6 weeks [1]. CU is further subdivided into chronic inducible urticaria (CIndU) and chronic spontaneous urticaria (CSU). CSU affects all age groups but has its highest incidence in the working‐age population (20–40 years), where it substantially impairs quality of life, affecting daily activities, sleep, and psychological well‐being [2].

The etiology of CSU is often elusive, though an autoimmune basis is suspected in 30%–50% of cases. This autoimmune pathogenesis is broadly classified into two distinct endotypes [1]. Type I autoimmune (autoallergic) CSU is characterized by the presence of IgE autoantibodies directed against various autoantigens, such as thyroid peroxidase (TPO). Clinically, Type I patients often present with higher total serum IgE levels, a strong association with other atopic comorbidities, and typically exhibit a rapid and favorable response to antihistamine or anti‐IgE therapy [3]. In contrast, Type IIb autoimmune CSU is mediated by IgG autoantibodies that directly target either IgE or the high‐affinity IgE receptor (FcεRI) itself, leading to mast cell activation. These endotypes often present with different clinical and laboratory features; Type IIb CSU, for example, is frequently associated with higher disease severity, concomitant autoimmune diseases, a poor response to antihistamines or omalizumab, and a better response to cyclosporine [4, 5]. Management primarily relies on second‐generation H1‐antihistamines. However, many patients require dose escalation or adjunct therapies like omalizumab or cyclosporine, to achieve symptom control [1, 6].

Recent large‐scale genome‐wide association studies (GWAS) have begun to investigate the genetic associated risk and pathogenesis of CSU. These studies successfully identified several new risk loci (such as HLA‐DQA1 and PTPN22) and provided strong evidence that CSU pathogenesis shares a significant genetic overlap with autoimmune diseases, rather than atopic conditions [7, 8]. However, these studies, which successfully linked genetic factors to autoimmune‐related phenotypes, did not identify the genes for the primary effector receptors, *FCER1A* (encoded for Fc epsilon receptor I alpha) and *PTAFR* (encoded for platelet‐activating factor receptor), as significant susceptibility loci. This suggests that while genetic risk points to an upstream autoimmune dysregulation, clinical heterogeneity is likely driven by downstream transcriptional control of these key effector pathways, rather than by variation within the effector genes themselves. This pathogenic complexity is reflected in key biomarkers. Many studies have shown that the expression of *FCER*1A gene is elevated in CSU patients and can predict the response to omalizumab, an anti‐IgE therapy [9, 10]. In addition to the classic IgE‐mediated pathway, other receptors are implicated in CSU pathogenesis. Platelet‐activating factor (PAF) and its receptor (PAFR), encoded by the *PTAFR* gene, axis is involved in urticaria and other hypersensitivity reactions, with elevated PAF levels found in antihistamine‐refractory CSU patients [11, 12, 13]. These reports, therefore, revealed strong links between CSU disease activity and two key biomarkers: FcεRI protein expression and elevated serum PAF levels. This focus on protein expression and serum ligands, however, leaves a critical gap in our understanding, as it remains unknown whether these changes originate at the transcriptional level. For instance, it has not been determined if the elevated FcεRI protein on mast cell and basophils should be a direct result of increased *FCER*1A gene transcription. Similarly, while serum PAF (the ligand) is high, the corresponding gene expression of its receptor (*PTAFR*) on effector cells has not been characterized. Therefore, characterizing this transcriptional heterogeneity is a critical next step, as it holds the potential to explain the diverse clinical responses to therapy and guide more personalized treatment strategies.

Although, mast cells are the primary effector cells in the skin, its isolation could be an ideal protocol to gain and study their role in CSU pathogenesis. However, in vitro study requires skin biopsies and special isolation techniques, which is very invasive and difficult to acquire enough amount of mast cell to perform complete experiments. Basophils, which are readily accessible in peripheral blood and share many functional similarities with mast cells, serve as a practical and relevant surrogate. Both cell types express FcεRI and can release a similar array of inflammatory mediators upon activation [9, 14, 15]. We hypothesize that the transcription levels of *FCER*1A and *PTAFR* in basophils may correlate with disease severity. Furthermore, these gene expression profiles may serve as accessible and practical translational biomarkers to predict therapeutic response or to guide treatment. Therefore, this study aimed to quantify and compare the transcription profiles of *FCER*1A and *PTAFR* in peripheral blood basophils isolated from CSU patients with mild, moderate, and severe disease activity, and compared with healthy controls.

## 2 Methods

### 2.1 Study Design and Population

This cross‐sectional pilot study was approved by the Siriraj Institutional Review Board, Faculty of Medicine Siriraj Hospital, CoA No. Si 764/2022. We recruited 45 adult Thai patients (≥ 18 years) diagnosed with CSU at the Dermatology outpatient unit of Siriraj Hospital. Patients were stratified into three severity groups: mild (*n*  =  15), moderate (*n*  =  15), and severe (*n*  =  15), based on UAS7 and medical scores [15, 16, 17]. The UAS7 scores were derived from the cumulative daily wheal scores (ranging from 0 to 3) and pruritus intensity scores (ranging from 0 to 3) over a period of 7 consecutive days. The UAS7 severity levels categorize urticaria as mild (0–15 points), moderate (16–27 points), and severe (28–42 points). The scoring system determined weighted points for different medications: oral antihistamines (2 points for standard dose, 8 points for fourfold dose), systemic corticosteroids (< 11 mg, 5 points; 11–25 mg, 10 points; > 25 mg, 15 points), ciclosporin (8 points for > 3 mg/kg/day), hydroxychloroquine (6 points), and leukotriene receptor antagonists (2 points). However, the original medication scoring system does not cover omalizumab, which is included in the treatment guideline of urticaria. Therefore, the authors added an omalizumab score as eight points, which is the same score as ciclosporin. The cumulative medication score was calculated from summation of maximal scores of each medication the patients received since the start of treatment. The higher score indicated the higher disease severity. The CSU patients were also evaluated through immunological biomarkers (total IgE and anti‐FcεRI IgG) to determine endotype characteristics [3]. They were defined as Type I alone, when total IgE level was more than 100 kU/L, and as Type IIb alone, when anti‐FcεRI IgG level was more than 936.7 ng/mL [18]. If the levels of both markers in their sera were below the cut‐off, they were defined as undetermined/unknown; if they were above the cut‐off, they were defined as mixed type (Type I + IIb). A control group of 15 healthy adult volunteers with no history of urticaria, allergy, or autoimmune disease was also recruited. Written informed consent was obtained from all participants.

### 2.2 Blood Collection and Basophil Isolation

To reduce interference of medication to gene expression, the blood samples were collected from the CSU patients, who were able to omit oral corticosteroids (30 days), immunosuppressive drugs (30 days), omalizumab (5 months), and hydroxychloroquine (5 months) prior to blood donation [19, 20]. A 50 mL peripheral blood sample was collected from each participant into EDTA tubes and basophils were immediately isolated from whole blood using the EasySep Human Basophil Enrichment Kit (STEMCELL Technologies, Vancouver, Canada). The isolation kit was a negative selection immunomagnetic separation technique to minimize cell activation. The purity of the isolated basophils was assessed by flow cytometry using fluorescent‐labeled antibodies against basophil‐specific markers CCR3 (PE) and CD123 (PerCP‐Cy5.5).

### 2.3 RNA Extraction and cDNA Synthesis

Total RNA was extracted from the purified basophils using the All Prep DNA/RNA Mini Kit (Qiagen, Hilden, Germany). RNA quantity and quality were assessed, and the RNA was reverse‐transcribed into complementary DNA (cDNA) using a high‐capacity cDNA reverse transcription kit (Qiagen, Hilden, Germany). The resulting cDNA was stored at −80°C until use.

### 2.4 Real‐Time Quantitative PCR (RT‐qPCR)

The relative mRNA expression of *FCER*1A and *PTAFR* was quantified by RT‐qPCR using SYBR Green dye‐based chemistry on a real‐time PCR system. *GAPDH* was used as the housekeeping gene for normalization. The primer sequences used are listed in Table 1. The relative gene expression was calculated using the comparative Ct (2^−ΔΔCt^) method.

**TABLE 1:** Gene primers.

Transcript target	Primer direction	Oligonucleotide sequence (5′ → 3′)
*GAPDH*	Forward	TTCACCACCATGGAGAAGGC
	Reverse	GGCATGGACTGTGGTCATGA
*FCER1A*	Forward	AATGGCAGCCTTTCAGAAGA
	Reverse	CTCATAGTCCAGCTGCCACA
*PTAFR*	Forward	GCACCAACTGTGTCTTAGACCC
	Reverse	TGGCACAACCACTTCAGTGACC

*Note:* All primer sequences are written in the 5′ to 3′ direction. Primers for *FCER*1A, and *PTAFR* were designed using the Primer‐BLAST tool (NCBI). Primers for *GAPDH* were sourced from [21].

### 2.5 Statistical Analysis

Statistical analysis was performed using GraphPad Prism version 10. Normality distribution of data was performed. Parametric data was analyzed with ANOVA followed by Tukey's multiple comparison test to reveal adjusted *p* value. Non‐parametric data was done with Kruskal‐Wallis followed by Dunn's multiple comparisons test to reveal adjusted *p* value. Binomial data sets (gender and disease status) were analyzed with Barnard's or Fisher's exact test. Normally distributed data were presented as mean ± standard error of mean (SEM). Non‐normally distributed data were presented as median and interquartile range (IQR). To evaluate the relationship between molecular transcription and clinical severity, Spearman's rank correlation coefficient (*r*_*s*_) was calculated between the relative *FCER1A* and *PTAFR* mRNA expression and severity category for all CSU patients. A *p*‐value < 0.05 was considered statistically significant.

## 3 Results

### 3.1 Participant Demographics and Clinical Characteristics

A total of 60 individuals were enrolled: 45 CSU patients and 15 healthy controls. The demographic data are summarized in Table 2. 45 CSU patients were classified to be the mild, moderate, and severe CSU groups according to treatment history, UAS7 and MS. The age and gender distribution were comparable across all groups. All 45 CSU patients were in an active disease state at recruitment, with 22.2% being uncontrollable despite antihistamine treatment. Although tendency of number of uncontrollable cases went along with severity, statistical analysis was not significant. Furthermore, most uncontrollable cases were found within the severe CSU cohort (46.5%). These comparative characteristics ensured that potential confounding effects were minimized when analyzing CSU severity as well as healthy controls. UAS7 scores significantly differentiated the severity levels between the mild and moderate/severe groups, but not between the moderate and severe groups. Further analysis for immunological biomarkers (total serum IgE and anti‐FcεRI) revealed that only anti‐FcεRI IgG levels were significantly elevated in CSU patients compared to healthy controls. Although levels of these biomarkers trended upward with disease activity, these differences did not reach statistical significance among the three groups. The distribution of CSU endotypes varied with severity, and our cohort's determination revealed a distinct shift in endotype distribution as clinical severity increased. Although Type IIb alone and mixed type were dominant across groups, the proportion of CSU with mixed type increased with severity. Interestingly, in the severe CSU cohort, Type I alone was not present (0/15) in this subset.

**TABLE 2:** Demographic and clinical data of study participants.

Group (*n*)	Healthy (15)	Chronic spontaneous urticaria (CSU) (45)	*p* value	Mild CSU (15)	Moderate CSU (15)	Severe CSU (15)	*p* value
Gender, *n* (male: female)	3:12	5:40	0.337	1:14	3:12	1:14	0.650
Age, years (mean ± SEM)	36 ± 2.8	43 ± 2.1	0.305	46 ± 3.7	40 ± 3.6	44 ± 3.8	0.742^a^
							0.988^b^
							0.951^c^
Disease status, *n* (%)	NA	Active (10/45; 22.2% un‐controllable)	NA	Active (1/15; 6.7% un‐controllable)	Active (2/15; 13.3% un‐ controllable)	Active (7/15; 46.5% un‐ controllable)	0.744
UAS7, range; median (IQR)	NA	0–42; 14 (10, 20)	NA	0–17; 5 (2, 10)	2–26; 20 (10, 21)	7–42; 22 (14, 35)	0.0362^a^
							< 0.0001^b^
							0.6997^c^
MS, range; median (IQR)	NA	0–23; 2 (2, 8)	NA	0–8; 2 (2, 2)	2–8; 8 (2, 8)	0–23; 8 (2, 18)	0.539^a^
							0.242^b^
							0.620^c^
Total IgE, kU/L (range; median (IQR))	4.3–537.0; 45 (9.5113)	8.4–936.0; 73 (47, 118)	> 0.999	8.4–936.0; 68 (31, 102)	15.6–549.0; 73 (28, 163)	14.4–286.0; 149 (14, 286)	> 0.999^a^ ^,^ ^b^ ^,^ ^c^
Anti‐FceRI, ng/mL (range; median (IQR))	178.4–3147.0; 360.8 (272, 1035)	547.5–5345; 1191 (1035, 1589)	< 0.001	547.5–4445.0; 1093 (725.4, 1629)	730.1–2997.0; 1191 (823.7, 1714)	652.8–5345.0; 1589 (1104, 2236)	> 0.999^a^ ^,^ ^b^ ^,^ ^c^
Endotype	NA	Undetermined/unknown (9/45) 20.0%	NA	Undetermined/unknown (5/15) 33.3%	Undetermined/unknown (2/15) 13.3%	Undetermined/unknown (2/15) 13.3%	NA
		Type I alone (5/45) 11.1%		Type I alone (2/15) 13.3%	Type I alone (3/15) 20.0%	Type I alone (0/15) I 0.0%	
		Type IIb alone (17/45) 37.8%		Type IIb alone (6/15) 40.0%	Type IIb alone (6/15) 40.0%	Type IIb alone (5/15) 33.3%	
		Mixed (type I + IIb) (14/45) 31.1%		Mixed (type I + IIb) (2/15) 13.3%	Mixed (type I + IIb) (4/15) 26.7%	Mixed (type I + IIb) (8/15) 53.3%	

*Note:* Data are shown as ratio, mean ± SEM, or range and median (95% confidence interval) or number (%). Normality distribution of data was performed. Parametric data was analyzed with ANOVA followed by Tukey's multiple comparison test to reveal adjusted *p* value. Non‐parametric data was done with Kruskal‐Wallis followed by Dunn's multiple comparisons test to reveal adjusted *p* value. Binomial data sets (gender and disease status) were analyzed with Barnard's or Fisher's exact test. kU/L stands for kilo‐units per liter and NA stands for not available.

^a^ Comparison between mild and moderate.

^b^ Comparison between mild and severe.

^c^ Comparison between moderate and severe.

### 3.2 Purity of Isolated Basophils

**FIGURE 1:**
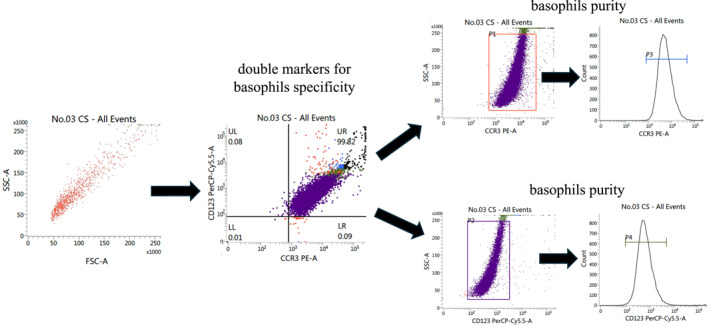
Flow cytometric gating strategy and purity assessment of human basophils isolated by negative selection. Representative flow cytometry analysis of basophils purified from peripheral blood mononuclear cells (PBMCs) of a patient with chronic spontaneous urticaria (CS) using a negative magnetic selection kit. (Left) The initial scatter profile of the total enriched cell fraction is shown by forward scatter (FSC‐A) versus side scatter (SSC‐A). (Middle) This population was subsequently analyzed for basophil‐specific surface marker expression, plotting CCR3‐PE (*x*‐axis) against CD123‐PerCP‐Cy5.5 (*y*‐axis). Basophils are identified as the double‐positive (CCR3^+^, CD123^+^) population in the upper‐right (UR) quadrant, demonstrating a purity of > 95% in this representative sample. (Right) Histograms confirm the high purity and uniform expression of CCR3 (top) and CD123 (bottom) within the isolated basophil population.

The negative selection method yielded a highly pure basophil population. Flow cytometry analysis confirmed an average purity of 99.46% ± 0.5% (Mean ± SD) based on CCR3 and CD123 co‐expression, ensuring that the subsequent gene expression analysis was specific to basophils (Figure 1).

### 3.3 Gene Expression in CSU Patients Versus Healthy Controls

**FIGURE 2:**
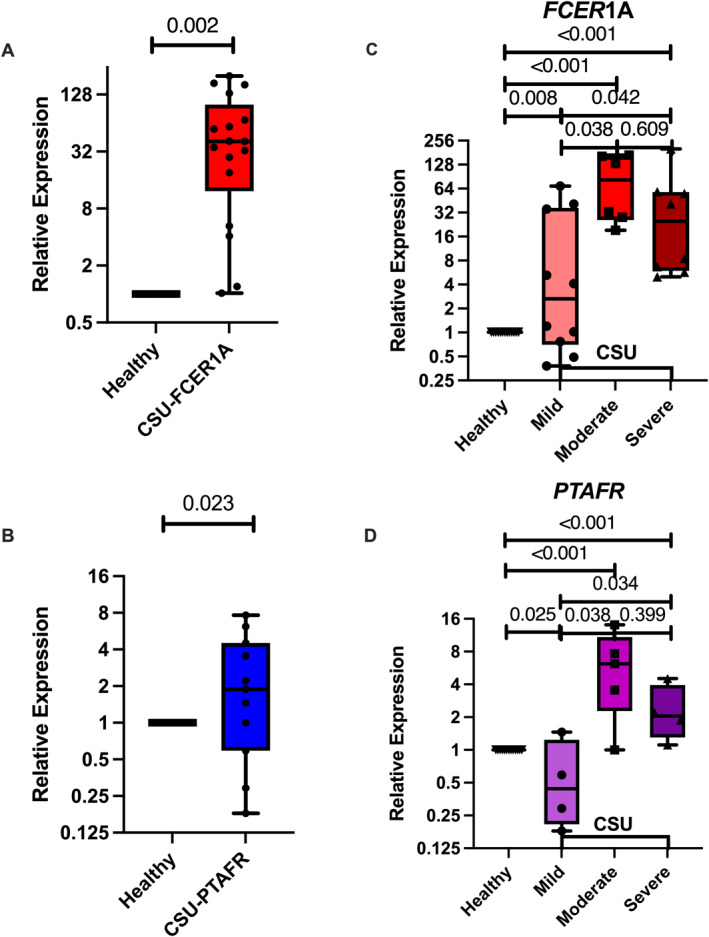
Relative mRNA expression of *FCER*1A and *PTAFR* in basophils isolated from healthy controls and across CSU severity groups. (A–B) Comparison of relative mRNA expression levels of (A) *FCER*1A and (B) *PTAFR* in peripheral blood basophils isolated from healthy controls (HC) and the total cohort of CSU patients. (C) Stratified analysis of *FCER*1A expression across HC and CSU severity subgroups (mild, moderate, and severe). Expression markedly increased with disease activity, especially in the moderate and severe groups. (D) *PTAFR* upregulation correlated with severity: Relative mRNA expression of the *PTAFR* gene among the same classified groups. Levels were markedly increased in moderate‐to‐severe CSU relative to both HC and the mild CSU group. Gene expression was measured using RT‐qPCR and normalized against the endogenous GAPDH housekeeping gene. Data were given as log2‐scale relative expression levels. Superimposed scatter‐box plots illustrated the median (central line) and the 5th–95th percentiles (interquartile range). Statistical comparisons among groups were conducted utilizing a two‐tailed Mann‐Whitney *U* test. The exact *p* value was above the line of comparison, and a value below 0.05 indicated statistical significance.

When all CSU patients were analyzed as a single cohort, the relative expression of *FCER*1A and *PTAFR* was significantly higher than in healthy controls (Figure 2A,B).

#### 3.3.1 *FCER*1A Expression

*FCER*1A mRNA expression was significantly elevated in all three CSU severity groups compared to healthy controls. Furthermore, expression levels increased with disease severity, with the moderate and severe groups showing significantly higher expression than the mild group (Figure 2C). There was no significant difference between the moderate and severe groups. Spearman's rank correlation revealed a significant positive correlation between relative *FCER1A* gene expression and severity categories (*r*_*s*_ = 0.4319, *p* value = 0.0351).

#### 3.3.2 *PTAFR* Expression

*PTAFR* mRNA expression was significantly higher in the moderate and severe CSU groups compared to both healthy controls and the mild CSU group. Interestingly, the mild CSU group showed significantly lower PAFR expression than the healthy controls (Figure 2D). A significant positive correlation was also observed between *PTAFR* mRNA expression and disease severity (*r*_*s*_ = 0.7794, *p* value = 0.0317).

## 4 Discussion

This study provides the first detailed analysis of *FCER*1A and *PTAFR* gene expression in basophils across distinct severity of CSU. Our findings reveal that the expression of these key pathogenic genes in highly relevant cell type correlates with disease severity, offering new insights into the molecular basis of CSU and identifying potential biomarkers. Assessment of gene expression provides a more molecular perspective compared to studies focusing on systemic biomarkers, such as serum C‐reactive protein or total IgE levels, which are often discussed in the context of CSU [22, 23]. This specificity allows for an investigation of cell‐intrinsic dysregulations that may be more directly involved in the effector mechanisms of CSU, such as wheal formation and pruritus, rather than reflecting broad systemic inflammation.

The study population exhibited a strong female predominance consistent with the known epidemiology pattern of CSU [24, 25]. A higher prevalence of female CSU was noted across various geographical regions [24, 26]. Furthermore, our observation of a substantial preference for females is corroborated by a similar tendency for related autoimmune disorders and refractory symptoms [27]. Analysis of the clinical data in Table 2 reveals that although total IgE levels exhibited an upward trend corresponding with disease severity, this increase was not statistical significance and remained comparable to levels observed in healthy controls. Likewise, while anti‐FcεRIα IgG is a valid autoimmune biomarker for differentiating CSU patients from healthy controls, its levels were not significantly concordant with clinical severity in our CSU cohort, aligning with unpublished subgroup analysis from our previous study [18]. The insignificant levels of total IgE and anti‐FcεRI IgG did not correspond with the significant increase in *FCER*1A mRNA expression across the severity groups. It has been reported that serum IgE might affect FcεRI expression on basophil surfaces; however, it did not impact FCER1A mRNA expression [28, 29]. Collectively, the finding suggested that ligand‐induced effect may not influence *FCER*1A expression.

Therefore, the cohort was dedicatedly selected for a more constrained range in age, female predominance, total serum IgE levels, anti‐FceRI IgG to minimize heterogeneity for elucidating the influence of genetic factors. To guarantee that our results actually represented the genetic transcription of basophils, we minimized gene expression profiles from a mixture of PBMCs, which could have obscured the essential gene expression in CSU effector cells, through using high‐purity basophil separation (> 99.0%). We also utilized a negative selection approach to reduce noise activation during basophil isolation, thereby obtaining authentic molecular expression of the basophils.

Our results also showed a significant increase in *FCER*1A expression in basophils from CSU patients compared to healthy controls with even higher levels observed in patients with moderate and severe CSU. This pattern is consistent with several studies that have demonstrated increased *FCER*1A expression in allergic and autoimmune conditions, particularly in peripheral blood basophils, skin mast cells, and circulating hematopoietic progenitor cells. The significant increase of expression on these cells positively correlated with disease activity and might serve as a potential predictor for the therapeutic response [9, 30]. This molecular priming suggests that the increased sensitivity and lower activation threshold characteristic of severe, refractory CSU may be governed by the cellular internal transcriptional dysregulation, independently of the serum levels of total serum IgE and anti‐FcεRI. FcεRI is a critical receptor involved in IgE‐mediated mast cell and basophil activation, a key pathway in the pathophysiology of CSU. A higher number of FcεRI receptors on the cell surface provides more binding sites for both IgE and the IgG autoantibodies, which may contribute to low threshold leading to the release of histamine and other inflammatory mediators that drive urticarial symptoms [31, 32]. The amplification of the activation signal exacerbates the chronic inflammatory condition and severity observed in CSU [33]. These findings support the strong positive correlation between FcεRI expression and CSU severity.

Similarly, *PTAFR* expression correlated significantly with disease severity, with the highest levels in moderate and severe CSU. This is consistent with studies reporting elevated PAF levels in antihistamine‐refractory CSU, and the role of the PAF‐PAFR axis in inflammatory plasma leakage [13, 34, 35, 36]. Recent report revealed that excess of PAF could generate vicious cycle of inflammation (PAF‐IL‐4) that was IgE‐receptor independent and provide another explanation for refractory to anti‐histamine and anti‐IgE treatment in some patients (Type IIb autoimmunity) [37, 38, 39]. In addition, increased levels of PAF could predict anti‐histamine resistance in CSU patients [13]. This evidence suggests that the PAF pathway is significantly upregulated in more severe forms of the disease. Integrate our discovery that a high genetic background of *PTAFR* in basophils of moderate to severe CSU patients could serve as a noteworthy severity marker and consider targeting this pathway using specific antagonists for severe CSU, as utilized in other diseases [40].

**FIGURE 3:**
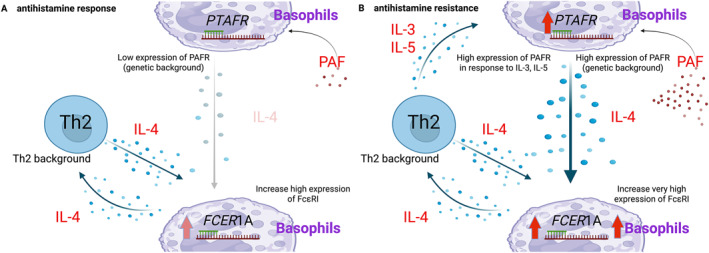
A schematic of the proposed immunological profiles in antihistamine responsive CSU (mild CSU); (A), and antihistamine resistant CSU (moderate‐severe CSU); (B). The illustration presents interaction between the genetic background of *PTAFR* and *FCER1*A in basophils, Th2 milieu and PAF‐IL‐4 axis. This figure was designed and illustrated using BioRender. Created in BioRender. Srinoulprasert, Y. (2026) https://BioRender.com/xuwljll and https://BioRender.com/epnki0d.

From our findings and previous PAF‐IL‐4 axis approach, we propose an immunological mechanism of antihistamine resistance (moderate to severe) CSU as illustrated in Figure 3. CSU presents distinct immunological profiles leading to mild to severe symptoms. According to clinical practice, mild CSU cases respond well to antihistamine treatment (antihistamine response), however, antihistamine treatment failure (antihistamine resistance) was found in moderate‐severe cases. In Mild CSU, Figure 3A, basophils are characterized by low expression of *PTAFR* with low PAF milieu making low IL‐4 production of PAF‐IL‐4 axis of vicious cycle. However, high expression of *FCER*1A could be regulated by IL‐4 of Th2 immune environment of CSU. On the contrary, moderate‐severe CSU, where the condition is driven by a more aggressive basophil phenotype, significantly high expression of *PTAFR* and high PAF milieu as illustrated in Figure 3B. This condition drives PAF‐IL‐4 vicious cycle to release IL‐4 merging with IL‐4 from Th2 environment, which can drive more expression of *FCER*1A [41, 42]. Very high expression of FcεRI basophils could have a high chance for anti‐FcεRI autoantibody as well as IgE cross linkage leading to enhance Th2 milieu via IL‐4 production loop. When Th2 conditions are present, IL‐3 and IL‐5 can promote increased expression of *PTAFR* in basophils through feedback mechanisms. A critical finding in moderate‐severe CSU is the establishment of a vicious cycle of PAF‐IL‐4 axis with the significantly high expression of *FCER*1A on basophils. This condition could create a very low activation threshold, leading to increase production of IL‐4. This IL‐4, in turn, enhances Th2 responses, prompting the production of IL‐3 and IL‐5. These cytokines then reactivate basophils, driving the further upregulation of *PTAFR* expression [43], thereby perpetuating the cycle of inflammation and contributing to the severity and refractory to antihistamine/anti‐IgE treatment. Therefore, anti‐PAF medications and anti‐PAFR biologics can be a promising alternative therapeutic strategy for patients with moderate‐to‐severe CSU, who are refractory to high‐dose antihistamines and anti‐IgE therapies [36]. Therefore, additional clinical studies are necessary to examine the effectiveness of addressing the PAF/PAFR axis in this difficult‐to‐treat population.

This study has some limitations. (1) The sample size for each subgroup was relatively small, which may limit the statistical power for some comparisons. (2) Single cohort of Thai patients. Even it may reduce confounding from genetic variability, future multicenter studies with larger cohorts or other ethnic groups are needed to validate these findings and ensure generalizability. Additionally, this study measured mRNA expression, which may not always directly translate to protein levels or functional activity. Future research should integrate parallel study with skin mast cell, proteomics and functional assays (such as basophil activation test) will be essential to fully validate and provide a more complete picture of the roles these receptors play in CSU.

## 5 Conclusion

In conclusion, this study demonstrates that the expression of *FCER*1A and *PTAFR* in peripheral blood basophils strongly correlates with the clinical severity of chronic spontaneous urticaria. Especially, expression of *PTAFR* could be a promising biomarker used to stratify patients, predict disease courses, and guide the selection of targeted therapies. Targeting the PAF/PAFR pathways offers rational and promising approach for the development of novel treatments for patients with moderate to severe CSU, particularly.

## Author Contributions

**Kevin Muliawan Soetanto:** investigation, validation, formal analysis, data curation. **Chattip Sripatumtong:** investigation, data curation, formal analysis, validation. **Teerapat Paringkarn:** data curation. **Nattha Angkoolpakdeekul:** data curation. **Kanokvalai Kulthanan:** conceptualization, methodology, validation, writing – original draft, writing – review and editing, resources, supervision. **Yuttana Srinoulprasert:** conceptualization, methodology, supervision, formal analysis, funding acquisition, writing – original draft, writing – review and editing, validation.

## Funding

This research was funded by the This work was supported by (i) Mahidol University, (ii) Thailand Science Research and Innovation (TSRI), and (iii) National Science, Research and Innovation Fund (NSRF), project ID 181610. The funding sponsor had no role in the study design; in the collection, analysis, or interpretation of data; in the writing of the manuscript; or in the decision to publish the results.

## Ethics Statement

The study protocol was reviewed and approved by the Siriraj Institutional Review Board (SIRB), Faculty of Medicine Siriraj Hospital, Mahidol University (Certificate of Approval No. CoA No. Si 764/2022). All research was conducted in strict adherence to the ethical principles outlined in the Declaration of Helsinki and the Good Clinical Practice (GCP) guidelines.

## Consent

Written informed consent was obtained from all participants prior to enrollment, blood sample collection, and any study‐related procedures.

## Conflicts of Interest

The authors declare no conflicts of interest.

## Data Availability

The data that support the findings of this study are available from the corresponding author, Yuttana Srinoulprasert (yuttana.sri@mahidol.ac.th), upon reasonable request.
